# Simultaneous Recordings of Neuronal Activities, Pup Calls, and Maternal Behaviors in Lactating Mice

**DOI:** 10.1523/ENEURO.0040-25.2026

**Published:** 2026-07-28

**Authors:** Yueling Zang, Jiechang Tang, Xia Wang, Shanshan Liang, Xiaowei Chen, Huanhuan Wang

**Affiliations:** ^1^School of Medicine, Chongqing University, Chongqing 400030, China; ^2^Chongqing Institute for Brain and Intelligence, Chongqing 400064, China; ^3^Brain Research Center, Third Military Medical University, Chongqing 400038, China

**Keywords:** auditory cortex, maternal behavior, photometric recording, pup calls, simultaneous recording

## Abstract

Maternal behaviors are crucial for the survival and well-being of mammalian offspring. While previous studies have implicated both subcortical and cortical areas, including the auditory cortex (AuCx), in the regulation of maternal behaviors, the real-time dynamics of AuCx neuronal activity in response to pup calls during maternal behavior remain unclear. Here, we developed an integrated system that enables simultaneous recording of neural activity, pup calls, and maternal behaviors in freely behaving lactating female mice. Using this system, we observed reliable activity in AuCx neurons specifically during the pup contact phase of retrieval, but not during other maternal behaviors (grooming, crouching, nest building). Pup calls alone elicited no significant population response in AuCx, as measured by fiber photometry. Furthermore, this activity during pup contact showed a lack of association with the number of pup calls. This study provides an efficient tool for studying neural activity and behavioral dynamics in freely behaving animals, offering opportunities to investigate the neural mechanisms underlying maternal behaviors.

## Significance Statement

Maternal behaviors are crucial for the survival and well-being of mammalian offspring. In this study, we developed an integrated system that enables simultaneous recording of neural activity, pup calls, and maternal behaviors in freely behaving lactating mice. Using this system, we observed reliable activities in auditory cortex (AuCx) neurons during pup retrieval. This study provides an efficient tool for studying neural activity and behavioral dynamics in freely behaving animals, offering new opportunities to investigate the neural mechanisms underlying maternal behaviors. Beyond the AuCx, the system could be adapted to investigate the roles of other cortical and deep brain regions in regulating maternal behaviors.

## Introduction

Across mammalian species, females exhibit elaborate maternal behaviors essential for offspring survival (Dulac et al., 2014; Kohl et al., 2017; Kohl and Dulac, 2018; Rogers and Bales, 2019; Servin-Barthet et al., 2023). Over recent decades, significant progress has been made in identifying the brain regions and cell types that govern maternal behaviors, primarily through studies on rodents such as rats, mice, voles, and hamsters as well as sheep, rabbits, and birds (Numan and Stolzenberg, 2009; Wu et al., 2014; Fang et al., 2018; Kohl et al., 2018; Autry et al., 2021). In rodents, maternal behaviors include approaching and retrieving pups displaced from the nest, crouching over pups to provide warmth and nutrition, and grooming or licking them. Maternal behavior is coordinated by an integrated network of cortical and subcortical regions that process sensory, motivational, and hormonal cues. Sensory systems, including the central olfactory pathway and auditory cortex (AuCx), are essential for detecting infant-derived signals. Subcortical regions, such as the medial preoptic area and the paraventricular nucleus (PVN) of the hypothalamus, regulate hormonal influences (e.g., estrogen, oxytocin) that are critical for the initiation and maintenance of maternal care. The ventral tegmental area supports motivated aspects of caregiving through dopaminergic reward pathways, while the periaqueductal gray modulates motor output for maternal interactions (Cohen et al., 2011; Kaplan et al., 2025).

In mice, vocalizations serve as key communication cues between pups and adult caregivers. Ultrasonic distress calls, emitted by isolated pups, prompt experienced dams to retrieve the vocalizing pup and return it to the nest (Ehret, 2005; Lecca et al., 2023). Like other sounds, pup calls are processed by the auditory system. The AuCx consists of the primary AuCx (A1) and secondary AuCx (A2). A1 mediates initial sound processing, whereas A2 and the temporal association area (TeA), part of the broader auditory temporal association cortex, serve as higher-order regions that integrate auditory input with other sensory modalities to support complex sound processing. The primary AuCx (A1) undergoes structural and functional changes due to plasticity during the transition to motherhood, enhancing its responsiveness to pup calls (Liu et al., 2006; Nelken et al., 2007; Galindo-Leon et al., 2009; Cohen et al., 2011; Marlin et al., 2015; Shepard et al., 2016; Tasaka et al., 2018). The TeA showed strong activation when dams were exposed to pup ultrasonic vocalizations (Tasaka et al., 2020). Furthermore, the strong A1-to-TeA connectivity suggested an important role of the AuCx in encoding pup calls during motherhood (Tasaka et al., 2020). In parallel, seminal work by Valtcheva and colleagues identified a noncanonical auditory circuit that pup distress calls are primarily processed in the inferior colliculus rather than the AuCx. These signals are relayed to the posterior intralaminar nucleus of the thalamus (PIL), which directly innervates oxytocin-producing neurons in the PVN. This subcortical pathway promotes oxytocin release, a key maternal hormone, thereby facilitating maternal behavior (Valtcheva et al., 2023). However, it remains unclear whether maternal recognition of auditory cues, such as pup calls, relies more on canonical auditory pathways or these noncanonical circuits. Addressing this gap requires an integrated device capable of simultaneously recording pup calls, neural activity in the AuCx, and maternal behavioral events.

Mouse pup calls are typically ultrasonic distress vocalizations with a frequency range of 40–80 kHz (Perkel et al., 2011; Fonseca et al., 2021; Li et al., 2024). Previous studies using two-photon microscopy have identified a subset of neurons in the AuCx that specifically respond to pup calls. However, two-photon imaging poses experimental challenges in freely moving animals, such as lactating mice, limiting its utility in exploring the relationship between pup calls, neural activity, and maternal behaviors. In the last decade, photometric recording techniques have emerged as a simple yet effective method for recording neural activity in freely behaving animals (Qin et al., 2018, 2022). Fiber photometry offers several advantages for studying such processes: it is compatible with naturalistic behavior due to minimal movement constraints; it enables efficient signal detection from deep brain regions with minimal scattering, and it supports stable chronic recordings over extended periods, allowing longitudinal assessment of neural plasticity and learning. Taken together, fiber photometry offers more advantage over two-photon microscopy in the lactating mice.

In this study, we developed an integrated system that combines audio recording, video recording, and neural activity monitoring within the home cages of lactating mice and their pups. Using this system, we successfully recorded neural activity and pup calls during pup retrieval events.

## Materials and Methods

### Animals

All experimental procedures were approved by the Third Military Medical University Animal Care and Use Laboratory Animal Welfare and Ethics Committee of the Third Military Medical University protocols. Adult female C57BL/6J mice (3–5 months old) were used for all recording and behavioral experiments. Mice were housed under a 12 h light/dark cycle (8 P.M. to 8 A.M. light) with *ad libitum* access to food and water. Mice were allowed to mate naturally through cohousing with sexually experienced C57BL/6J males, without the use of assisted reproductive techniques. Behavioral experiments were performed at the end of the light cycle of the mice. The animals used in this study were all primiparous. Females were housed individually following fiber implantation and remained singly housed until the end of the experiments.

### Stereotactic surgery

Mice were anesthetized with 1–1.5% isoflurane in oxygen and positioned in a stereotactic head frame over a heating pad maintained at 37–38°C. A small craniotomy (0.5 × 0.5 mm) was performed above the left AuCx at coordinates −3.00 mm anteroposterior (AP) and −3.80 mm mediolateral (ML) from the bregma using a dental drill after removing the overlying skin. pAAV2/9-hSyn-GCaMP6f-WPRE-pA (2.20 × 10^13^ vg/ml; OBiO Technology) was injected unilaterally into the left AuCx (AP, −3.00 mm; ML, −3.80 mm; DV, −1.5 mm; ∠22°) using a glass micropipette with a tip diameter of 15–20 µm. The AAV injection was targeted to the left AuCx based on prior evidence of functional lateralization in the processing of pup vocalizations (Marlin et al., 2015). A total of 150 nl of virus solution was injected at a rate of ∼20 nl/min. The micropipette was left in place for 5 min postinjection to prevent backflow. The craniotomy was sealed with tissue glue (Vetbond, 3M Animal Care Products). Experiments began 3 weeks after surgery to allow sufficient viral expression.

### Fiber photometry setup

**Figure 1.**
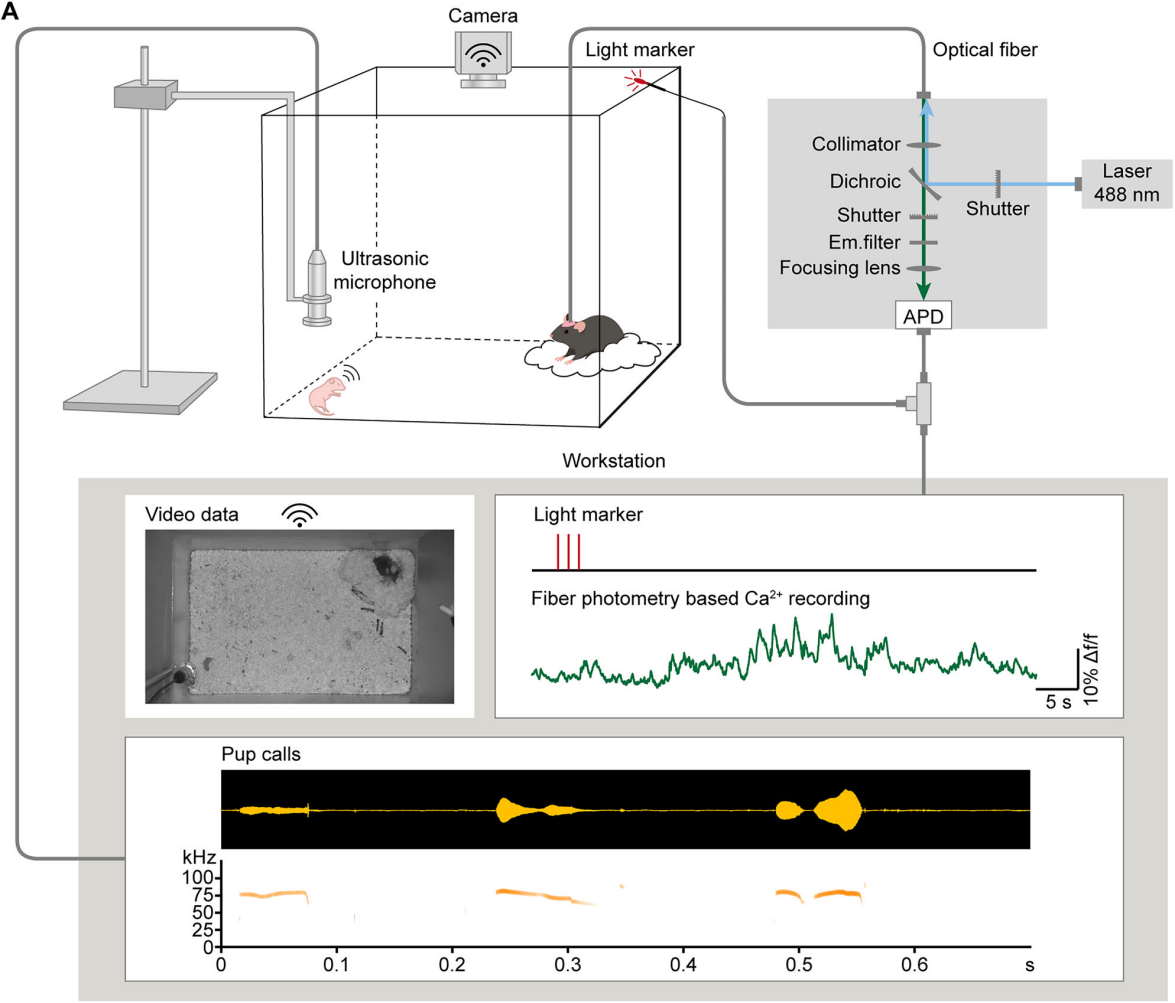
Schematic representation of the device for simultaneous recording of pup calls, neural activity, and maternal behavior during pup retrieval. The system integrates synchronized overhead video imaging (visible and infrared light), ultrasonic audio recording, and fiber photometry to monitor Ca^2^^+^ activity in the AuCx of freely behaving lactating mice. Extended Data Figure 1-1 supports this figure.

A custom-built fiber photometry system was used to record neural Ca^2^^+^ signals (Fig. 1*A*), which has been shown before (Zhang et al., 2017; Qin et al., 2018, 2022). Mice injected with GCaMP6f in the AuCx were anesthetized with 1–1.2% isoflurane and secured in a stereotactic head frame. A 200-µm-diameter optical fiber (NA 0.48; Doric Lenses, MFP_200/230/900-0.48) was inserted ∼100 µm above the A1 and attached to a short cannula (ID, 0.51 mm, and OD, 0.82 mm), with the fiber tip extending ∼2.5 mm beyond the cannula. The AuCx was excited at 488 nm, and fluorescence signals from the GCaMP6f indicator were captured by an avalanche photodiode (Si-APD, S2382, Hamamatsu Photonics) at a sampling rate of 2,000 Hz. Neural Ca^2^^+^ transients and behavioral video recordings were synchronized offline using the light markers (the flash of light-emitting diode, LED).

10.1523/ENEURO.0040-25.2026.f1-1 Figure 1-1 Schematic of pup calls analysis. Raw data (***A***) of pup calls obtained from Avisoft and the corresponding cleaned, classified output (***B***) generated by VocalMat. *Figure Contributions:* Yueling Zang, Jiechang Tang made the experimental device. Download Figure 1-1, TIF file.

### Audio acquisition

Pup calls were recorded using an ultrasonic microphone (CM16/CMPA; Avisoft Bioacoustics) connected to an UltraSoundGate 116H recording interface (Avisoft Bioacoustics) and positioned 15 cm above the isolated pup (Fig. 1*A*). Audio recordings were captured at a sampling rate of 250 kHz using the Avisoft RECORDER software and analyzed in VocalMat as described previously (Fonseca et al., 2021). Raw pup calls were recorded in .wav format with Avisoft and subsequently processed using the VocalMat package, which automatically detects and classifies ultrasonic vocalizations. The resulting output provides an Excel file containing the timing and classification of each call, enabling precise quantification of pup calls within any defined period (Extended Data Fig. 1-1). Pup calls (40–80 kHz) were distinguished from wriggling and adult calls based on acoustic features and behavioral context. Wriggling calls were identified by their low-frequency range (4–20 kHz) allowing for clear spectral separation from high-frequency pup calls (40–80 kHz; Geissler and Ehret, 2002; Ehret, 2005). Adult calls are rare without social interaction or aversive stimuli (Chen et al., 2021; Gan-Or and London, 2023; Xiao et al., 2023), neither of which were present in our paradigm. Based on this, we determined that the audio recordings contained no wriggling calls or adult calls. The audio acquisition system was synchronized with the fiber photometry setup for simultaneous recordings using the Quicker software.

### Behavioral experiments

Behavioral experiments were conducted in a semi-dark room under infrared illumination. To examine the interplay between behavioral events, neural activity, and pup call patterns driving maternal behavior in lactating mice, we developed a system for continuous monitoring. This system integrates synchronized overhead video recordings (visible light during the day and infrared at night), ultrasonic audio recordings, and fiber photometric recordings of neural activity (Fig. 1*A*). Videos were recorded from the top of the home cage using a synchronized camera system (Canon XA-25) at 25 frames/s (Fig. 1*A*). Specifically, synchronization was achieved by using a dedicated LED light marker from the fiber photometry system, positioned within the camera's field of view. A single TTL trigger pulse generated by Quicker simultaneously initiated data acquisition across all systems, ensuring a shared start time and common zero point. Both the LED marker and Avisoft software were triggered through the Quicker software. This setup provided a continuous, precise temporal reference: the photometry LED signal visible in the behavioral video enabled post hoc alignment of pup calls (Avisoft), neural calcium signals (fiber photometry), and video frames with millisecond accuracy. Lactating mice on postnatal days (PND; PND4–PND7) housed with their pups were tested in the home cage (a behavioral arena 55 × 42 × 27 cm) containing bedding and nesting materials. Females were prescreened to confirm maternal behaviors. Each female was presented with five pups scattered across the home cage, and only those that retrieved all pups to the nest within 10 min were included in subsequent experiments. This criterion is well established in the previous literature (Fang et al., 2018) and ensures inclusion of animals capable of performing the behavior of interest. Females displaying nonmaternal behaviors were excluded. A total of 11 lactating mice (52 trials) which retrieved spontaneously were recorded.

During testing, pups were temporarily separated from the dam for 2 min and were placed in a clean, glass cup preheated to 35°C using a heating pad. A single pup was then placed in the corner opposite the nest zone, and the dam was given 2 min to retrieve the pup. Pup retrieval was defined as the process of the lactating mice transporting the displaced pup back to the nest. If the pup was not retrieved, the trial was scored as a failure, and the pup was removed. The experiment was repeated with a different pup. Each mouse performed 4–6 trials per day during the behavioral testing phases. Approaching was defined as the dam initiates directed movement toward a displaced pup, pup contact as the moment the dam makes physical contact with the pup, and “Back to nest” as the interval between lifting the pup and returning it to the nest. Behavioral annotations were performed using the MotionCheck software on MATLAB (MathWorks), synchronized with neural activity recordings and audio data which has been shown before (Zhang et al., 2017).

### Histological imaging

At the terminal endpoints, mice were transcardially perfused with 4% paraformaldehyde to verify viral expression and optical fiber placement. Brains were postfixed overnight in 4% paraformaldehyde at 4°C, then embedded in resin embedding agent (Sakura; Tissue-Tek Optimal Cutting Temperature Compound), and sectioned (50 µm) using a cryostat. Sections were mounted on positively charged slides and stained with 4′,6-diamidino-2-phenylindole (1:2,000, Beyotime Biotechnology) for 10 min. Confocal images were acquired using a Leica TCS SP8 microscope and processed with the LAS AF software (Leica Microsystems).

### Data analysis and statistics

Calcium (Ca^2^^+^) transients were analyzed as described previously (Zhang et al., 2017; Qin et al., 2018). Fluorescence changes (Δ*F*/*F*) were calculated using the formula: Δ*F*/*F* = (*F − F*_baseline_)/*F*_baseline_, where *F*_baseline_ was the baseline fluorescence intensity during the recording. A Ca^2+^ transient was identified as a signal when the amplitude exceeded three times the standard deviation above the baseline. The baseline period was defined as a quiet, undisturbed interval when the dam was not engaged in maternal behaviors. The baseline was typically set at 500 ms prior to the behavior onset, ensuring a stable and representative reference signal for normalization.

Pup calls (40–80 kHz) were extracted using the VocalMat software (Fonseca et al., 2021). The number of pup calls during specific phases of maternal behavior was quantified by VocalMat. Statistical data are presented as mean ± SEM in the figures.

Statistical tests were performed in the MATLAB software. Comparisons of the number of pup calls across different behavioral phases (Fig. 3*D*), AuCx Ca^2^^+^ activity during pup contact events across postnatal days (Fig. 4*D*), and AuCx Ca^2^^+^ activity during pup calls (Extended Data Fig. 4-1*A*) were performed using the nonparametric two-sided Wilcoxon signed-rank test. AuCx Ca^2^^+^ activity across different maternal behaviors (Fig. 5*E*) was compared using the two-sided Wilcoxon rank-sum test*.* Pearson’s correlation was used to assess the relationship between AuCx activation and the number of pup calls during pup contact in Extended Data Figure 4-1*B*. Multiple comparisons were corrected by FDR adjustment (Benjamini–Hochberg method). All sample sizes, number of trials, and precision measures (mean, SEM) are provided in the figure legends.

## Results

### Simultaneous monitoring of neural activity, pup calls, and maternal behavior

**Figure 2.**
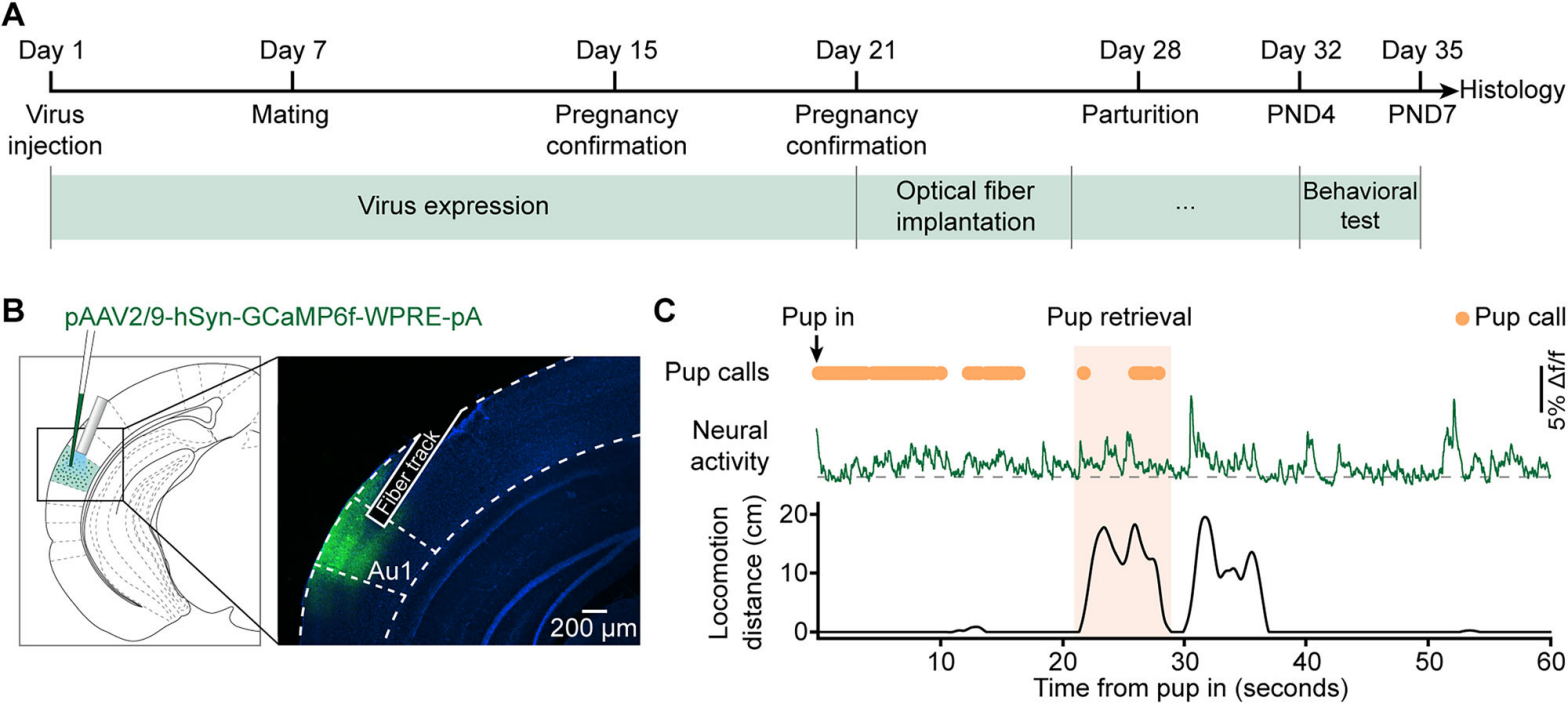
Simultaneous recording of pup calls, neural activity, and maternal behavior during pup retrieval. ***A***, Schematic of the experimental protocol for recording pup calls, neural activity, and maternal behavior in lactating mice. ***B***, Representative histological image showing GCaMP6f expression in the AuCx neurons and the placement of the fiber optic for photometric recordings. ***C***, Example traces showing synchronized recordings of pup calls (orange trace), neural Ca^2^^+^ activity (green trace), and maternal behavior events (black trace) during a 60 s pup retrieval session.

To investigate the role of auditory circuits in responding to pup call stimuli during maternal behavior, we expressed GCaMP6f, a genetically encoded fluorescent Ca^2^^+^ indicator, unilaterally in the AuCx (Fig. 2*A*). Histological analysis confirmed that GCaMP6f expression was localized to AuCx cells (Fig. 2*B*). Figure 2*C* presents an example of a 60 s recording, showing synchronized traces of Ca^2^^+^ signals (green trace), pup calls (orange trace), and behavioral events (black trace).

### Behavioral details of pup retrieval

**Figure 3.**
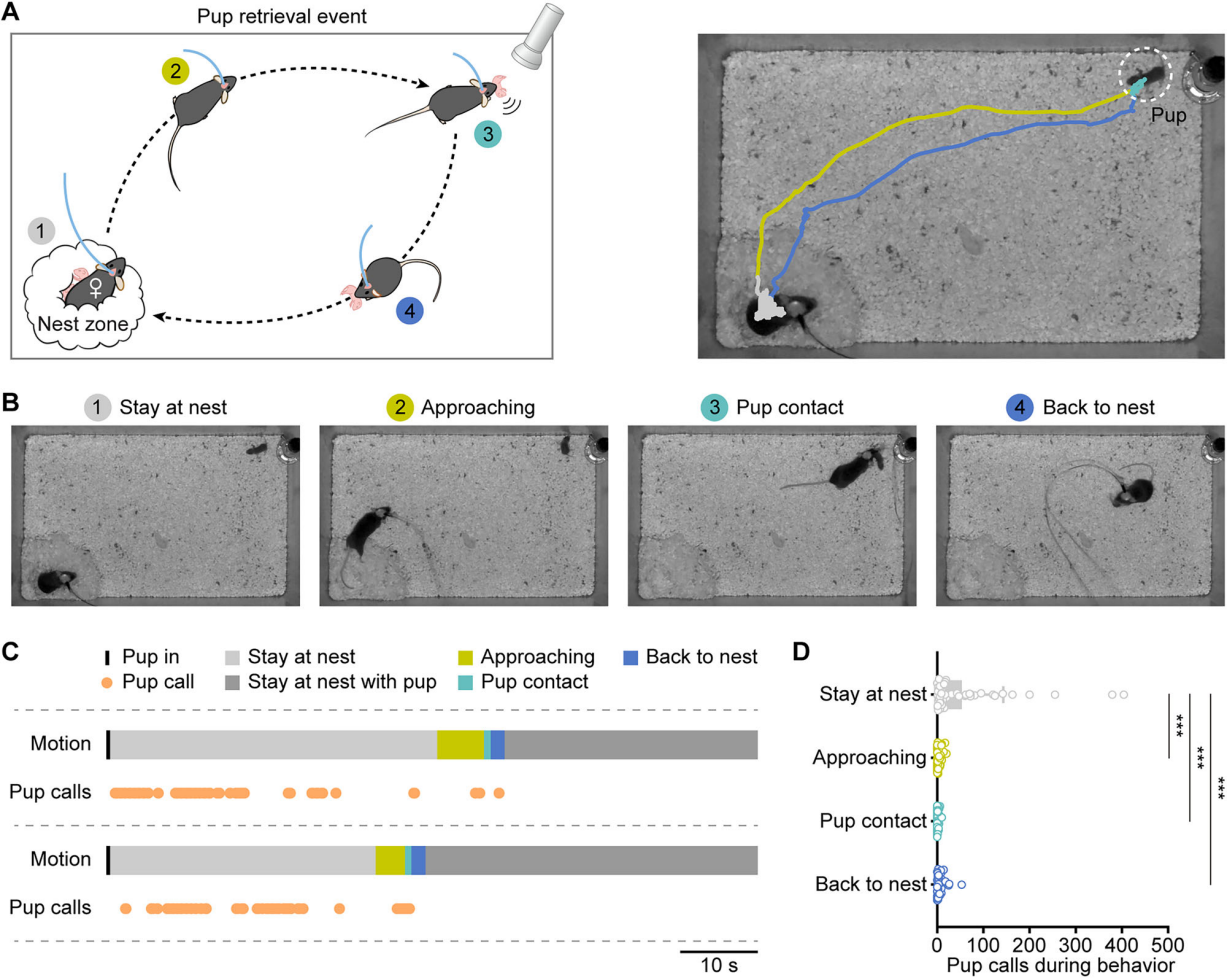
Pup calls and pup retrieval behaviors. ***A***, Left, Diagram illustrating the phases of a pup retrieval event, with each phase (Approaching, Pup contact, and Back to nest) indicated by a distinct color. White arrows denote the pup's location. Right, Example trace showing the temporal progression of behaviors during a single pup retrieval event. ***B***, Behavioral sequence observed after pup introduction. ***C***, Ethograms showing manually classified maternal behaviors aligned to the timing of pup call emissions during pup retrieval events. ***D***, Quantification of pup calls emitted during different behavioral phases of the lactating mice (*n* = 50 trials from 10 mice). Pup calls (mean ± SEM) were Stay at nest, 53.86 ± 12.64; Approaching, 3.40 ± 0.64; Pup contact, 1.43 ± 0.29; Back to nest, 5.86 ± 1.33. Stay at nest versus Approaching, *p* = 8.88 × 10^−8^; Stay at nest versus Pup contact, *p* = 1.69 × 10^−8^; Stay at nest versus Back to nest, *p* = 2.61 × 10^−7^; two-sided Wilcoxon signed-rank test, FDR corrected, ***p < 0.001.

Our study focused on pup retrieval behavior, as illustrated in Figure 3*A*. We quantified the duration of each phase (Fig. 3*B*) and observed that most dams initiated the approaching phase within 36.63 ± 7.25 s (*n* = 50 trials from 11 mice) after pup introduction, triggering the onset of pup retrieval.

### Pup calls and maternal behavior

We next analyzed the number of pup calls emitted during different stages of pup retrieval. Figure 3*C* shows representative traces of manually classified behaviors aligned to pup call emission. Notably, the number of pup calls during the “Stay at nest” phase was significantly higher than during other behaviors (Fig. 3*D*; two-sided Wilcoxon signed-rank test, FDR corrected, ***p < 0.001), indicating that pup displacement from the nest triggers distress calls, which strongly elicit maternal responses such as retrieval. We found that the probability distribution of pup call-related approaching was 48.45 ± 5.08% (*n* = 35 trials from 10 mice) indicating additional sensory cues from pups may involve in triggering the approach.

### AuCx neural activity during pup retrieval

**Figure 4.**
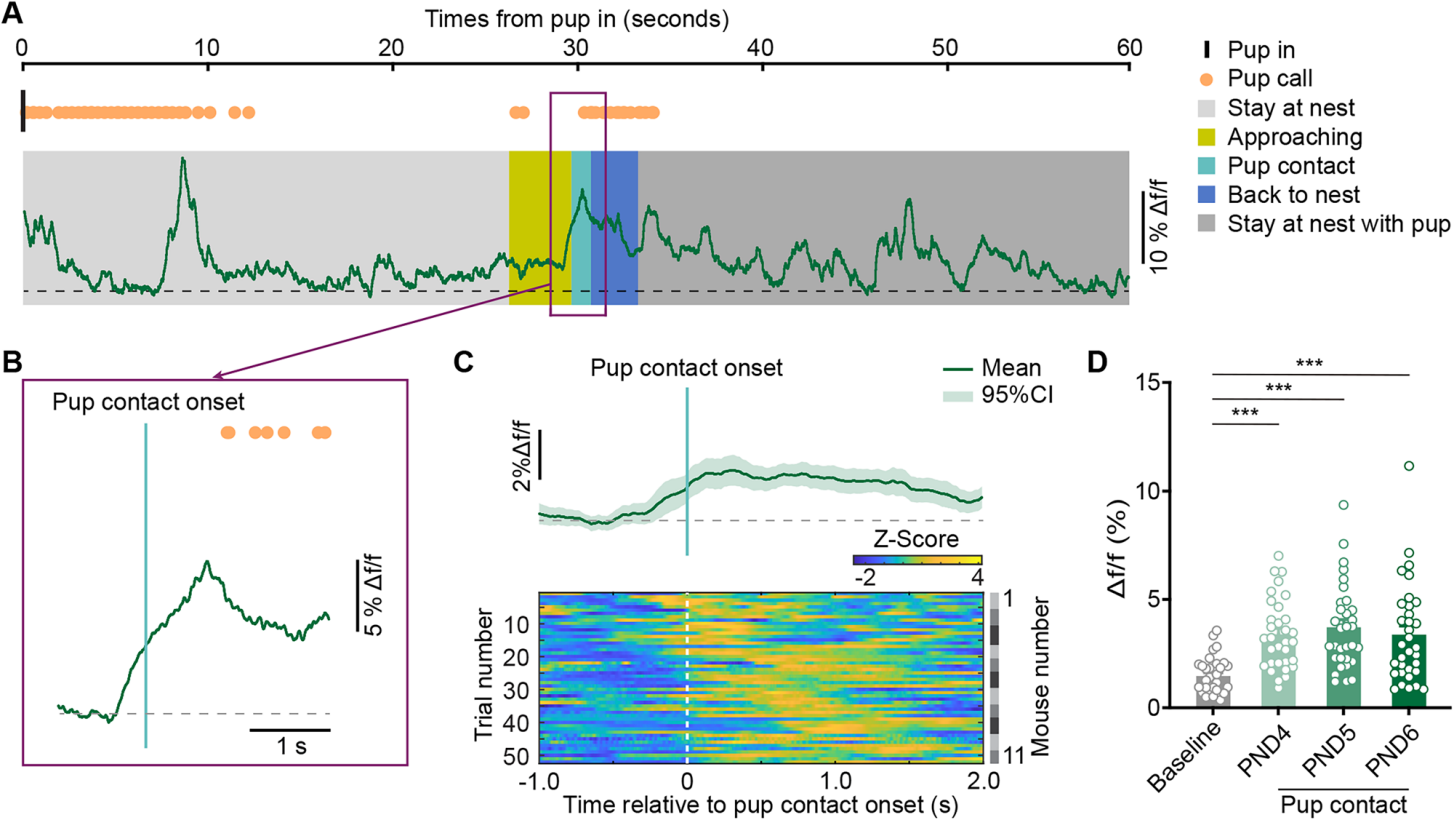
AuCx neuron activity correlates with pup retrieval. ***A***, Representative fiber photometry trace of AuCx neural Ca^2^^+^ activity synchronized with ultrasonic audio recordings during pup retrieval. ***B***, Zoomed in of the boxed area (purple) in ***A*** showing an example trace of Ca^2+^ transients recorded in the primary AuCx (***A***1) during the onset of pup contact. ***C***, Plot of Ca^2+^ transients aligned to pup contact onset (top), showing consistent increases in Ca^2+^ activity (*n* = 52 trials, 11 mice). Shaded areas represent the confidence intervals. Heatmaps of individual recording traces aligned to pup contact event onsets (bottom), illustrating variability and intensity of neural activity. ***D***, Quantification of amplitudes of pup contact-related Ca^2+^ transients (*n* = 32 trials, 7 mice). Baseline versus Pup contact (PND4), *p* = 1.61 × 10^−4^; Baseline versus Pup contact (PND5), *p* = 0.67 × 10^−4^, Baseline versus Pup contact (PND6), *p* = 2.94 × 10^−4^; two-sided Wilcoxon signed-rank test, FDR corrected, ***p < 0.001. Extended Data Figure 4-1 supports this figure.

**Figure 5.**
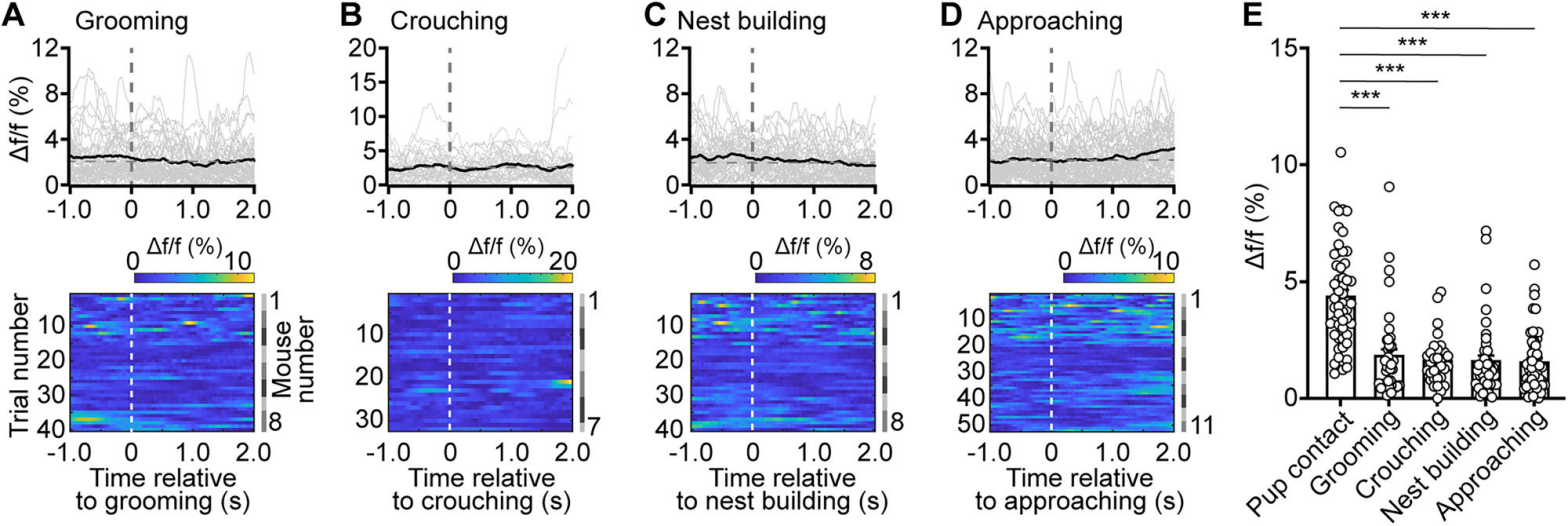
AuCx neuron activity in other maternal behaviors. ***A–D***, Plot of Ca^2+^ transients aligned to the onset (dashed lines) of grooming (***A***), crouching (***B***), nest building (***C***), and approaching (***D***). Top, Overlay of all trials [grooming (***A***), *n* = 40 trials, 8 mice; crouching (***B***), *n* = 32 trials, 7 mice; nest building (***C***), *n* = 40 trials, 8 mice; approaching (***D***), *n* = 52 trials, 11 mice]. Gray traces, individual trials; black, mean. Bottom, Heatmaps of individual Ca^2+^ transients aligned to onsets of grooming (***A***), crouching (***B***), nest building (***C***), and approaching (***D***). ***E***, Quantification of amplitudes of Ca^2+^ transients in different maternal behaviors. Pup contact versus Grooming, *p* = 1.50 × 10^−8^; Pup contact versus Crouching, *p* = 7.75 × 10^−9^; Pup contact versus Nest building, *p* = 1.60 × 10^−9^; Pup contact versus Approaching, *p* = 1.40 × 10^−10^; two-sided Wilcoxon rank-sum test, FDR corrected, ***p < 0.001.

We conducted photometric recordings of lactating mice during interactions with their pups, synchronized with pup call recordings (Fig. 4*A*). During pup contact events, we observed significant increases in Ca^2^^+^ transients at both the individual event level and averaged across animals (Fig. 4*B,C*; two-sided Wilcoxon signed-rank test, FDR corrected, ***p < 0.001). This effect was consistent across pup PND4–PND6 when compared with baseline (Fig. 4*D*; two-sided Wilcoxon signed-rank test, FDR corrected, ***p < 0.001). In contrast, pup calls themselves elicited little change in AuCx Ca^2^^+^ activity (Extended Data Fig. 4-1*A*; two-sided Wilcoxon signed-rank test, *p* = 0.396). Also, we found no significant correlation between AuCx activation and the number of pup calls during pup contact (Extended Data Fig. 4-1*B*; Pearson’s correlation; *r* = 0.1691; *p* = 0.2505). We next examined AuCx activation during other maternal behaviors, including grooming, crouching, and nest building. While the dam was grooming or crouching the pups or gathering nesting materials, the Ca^2+^ activity was more variable and not significantly changed across animals (Fig. 5*A–C*; two-sided Wilcoxon signed-rank test, FDR corrected, *p* = 0.629, 0.695, 0.162). We observed 53 grooming bouts (total duration, 719 s; mean, 10.6 ± 2.87 bouts/session; mean bout duration, 13.57 ± 1.79 s) and 34 crouching bouts (total duration, 662 s; mean, 6.8 ± 3.17 bouts/session; mean bout duration, 19.47 ± 2.44 s), each derived from five animals across five sessions, and 40 nest building bouts (total duration, 923 s; mean, 10.0 ± 1.78 bouts/session; mean bout duration, 20.75 ± 1.53 s) derived from four animals across four sessions. During pup approach, the amplitude of Ca^2^^+^ signals either remained unchanged or showed slight increases (Fig. 5*D*; two-sided Wilcoxon signed-rank test, FDR corrected, *p* = 0.856). Together, these results indicate that AuCx cells are preferentially activated during pup retrieval (pup contact), but not during other maternal behaviors (Fig. 5*E*; two-sided Wilcoxon rank-sum test, FDR corrected, ***p < 0.001).

10.1523/ENEURO.0040-25.2026.f4-1 Figure 4-1 No significant AuCx responses to pup calls. ***A***, Plot of Ca^2+^ transients aligned to the pup call onset (red dashed lines) of AuCx cells (n = 49 trials, N = 10 mice). ***B***, No significant correlation between AuCx activation and the number of pup calls during pup contact (n = 48 trials, N = 10 mice). *Figure Contributions:* Huanhuan Wang, Yueling Zang performed the experiments; Jiechang Tang, Xia Wang, and Shanshan Liang analyzed the data. Download Figure 4-1, TIF file.

## Discussion

In this study, we developed an integrated device that integrates overhead video imaging of the home cage (under infrared light) with synchronized pup call recordings and photometric recordings. This system enabled simultaneous monitoring of neural activity, pup calls, and maternal behavior events in freely behaving lactating mice. The same system is also compatible with visible light conditions, which could be employed in future studies depending on specific experimental requirements.

We specifically focused on pup retrieval behavior, which we subdivided into three distinct phases: Approaching, Pup contact, and Back to nest. Our results revealed that Ca^2^^+^ activity in AuCx neurons significantly increased during pup retrieval (pup contact). This observation highlights the potential role of the AuCx in mediating maternal responses to direct pup interaction.

Compared with traditional in vivo electrophysiological recordings, photometric recordings offer significant advantages for monitoring population-level neural activity with high throughput. This approach also allows for targeted recordings of neural activity in specific subcellular structures (Qin et al., 2018, 2022). The integrated developed system provides an efficient platform for investigating circuit-level and cell type-specific mechanisms underlying maternal behavior. Previous studies have established systems primarily focused on dual-modal correlation with synchronizing self-generated adult vocalizations with concurrent neural activity or general behavioral state in the same animal (Chen et al., 2021; Winters et al., 2022; 2023; Gan-Or and London, 2023; Xiao et al., 2023). While highly effective for studying vocal production mechanisms, this setup is not readily adaptable for investigating the tripartite relationship among an external auditory stimulus, the neural response it evokes in a perceiving animal, and the resulting behavior. Our study applies and extends this technical approach specifically for investigating the maternal responses to pup USVs. We have optimized the system to achieve millisecond-level synchronization between acoustic stimulus, neural activity, and maternal behaviors. This stimulus–neural activity–behavior triad is uniquely suited to dissect how a biologically critical auditory cue from the offspring is processed by the maternal brain to drive a specific caregiving action.

Previous studies have shown that the AuCx undergoes plasticity in lactating mice, adapting to the demands of maternal care. Our findings further suggest that AuCx neurons exhibit functional specificity, with Ca^2+^ activity significantly increasing during pup retrieval (pup contact), but not during other maternal behaviors such as grooming, crouching, or nest building. It is important to note that the expression of maternal behaviors is highly context-dependent. While our study focused on pup retrieval, the experimental paradigm may not have provided the conditions necessary for the full expression of other maternal behaviors, such as grooming, crouching, and nest building. These behaviors typically depend on the presence and sustained tactile feedback from an entire litter in a stable home cage environment. Thus, the neural activity associated with these “other” behaviors in our study may reflect partial forms of these actions rather than their complete expression. This limitation should be considered when interpreting comparisons between neural signals during retrieval and those during other maternal behaviors. In addition, pup calls elicited no significant population response in AuCx as studied here by fiber photometry. Also, we found no significant correlation between AuCx activation and the number of pup calls during pup contact. The lack of pup call response in AuCx (including Au1 and AuD) contrasts with the previous study that reported neuronal responses to ultrasonic vocalizations in the AuCx (Tasaka et al., 2020). This difference could be due to methodological aspects (fiber photometry vs neuropixels probe recording). In vivo electrophysiology and fiber photometry offer advantages for neuroscience research. Electrophysiology provides direct, millisecond-resolution capture of neuronal electrical signals, including action potentials and local field potentials, making it ideal for high-frequency coding and network oscillations. Conversely, fiber photometry allows long-term, cell type-specific monitoring of population activity through genetically encoded indicators, albeit fundamentally limited by the slower kinetics of calcium dynamics. In essence, the choice represents a trade-off between the unparalleled temporal fidelity of electrophysiology and the targeted, long-term monitoring capability of fiber photometry. Using different recording locations could present another possibility. Histological analysis confirmed that the fiber was positioned primarily within the primary AuCx (A1), which may have limited detection of calcium signals from adjacent auditory regions more directly involved in pup retrieval. Future studies should target other AuCx subregions to determine whether neuronal activation differs across distinct phases of pup retrieval or during additional maternal behaviors. The primary objective of this study was to develop an integrated recording system to facilitate investigations into the mechanisms underlying pup call processing while placing limited emphasis on biological aspects. It is important to note that the prescreening of dams for the expression of maternal behavior prior to their inclusion in the study, while necessary to establish a baseline for our investigations, may have introduced a selection bias. Our results are therefore most directly applicable to dams that reliably exhibit maternal care. Future studies could extend this work by including a more diverse population.

Moreover, given the tonotopic organization of the AuCx, pup ultrasonic vocalizations (40–100 kHz) are preferentially processed in high-frequency subregions. Since our recordings were centered on A1, the fiber placement may not have been optimal for capturing neuronal populations most sharply tuned to these ultrasonic pup calls. This specificity underscores the heterogeneity of AuCx cell populations (Kalish et al., 2020; Gungor Aydin et al., 2024). A more refined characterization of these subpopulations could help identify neuron subsets directly involved in maternal behaviors. Future studies could employ single-nucleus RNA sequencing (snRNA-seq) to map the cellular diversity of AuCx neurons in adult mice. Combined with Cre-*loxP* genetic labeling, the system developed here could enable precise identification of molecularly defined neural populations and circuit elements that regulate maternal behavior.

In summary, we developed an integrated and efficient system for simultaneous pup call and photometric recordings during maternal behavior in lactating mice. This platform provides a simple method for studying neural activity and behavioral dynamics in freely behaving animals. Beyond the AuCx, the system could be adapted to investigate the roles of other cortical and deep brain regions in regulating maternal behaviors. While the current study focused on acute recordings, future implementations of this system could be scaled to enable continuous multiday neural and behavioral monitoring. With appropriate enhancements in data storage and computational resources, such extended recordings would facilitate the investigation of long-term processes including sleep cycles, learning consolidation, and social interaction dynamics.

## Synthesis

Reviewing Editor: Sam Golden, The University of Washington Decisions are customarily a result of the Reviewing Editor and the peer reviewers coming together and discussing their recommendations until a consensus is reached. When revisions are invited, a fact-based synthesis statement explaining their decision and outlining what is needed to prepare a revision will be listed below. The following reviewer(s) agreed to reveal their identity: NONE.

Dear Dr. Zang,

Thank you for your submitting a revised manuscript, which has now been re-reviewed. While the revised manuscript is improved, there are methodological and statistical detail that still need clarification prior to publication. Please see the below comments and provide a rebuttal and revised manuscript addressing all comments.

Comments:

The authors have improved the manuscript with the addition of a larger sample size, reduced redundancy across figures, and clearer language in response to several reviewer comments. While I found the authors generally responsive and the study to be of interest, there remain several outstanding concerns related to methodological clarity, statistical justification, and contextualization within the existing literature. Addressing the points below would substantially improve the transparency and interpretability of the work, particularly given the manuscript's emphasis on methodological innovation.

Major comments

1. Fiber photometry, USV recording, and behavioral video have been used together previously (e.g., DOI: 10.1126/sciadv.ade6992; DOI: 10.7554/eLife.85547; DOI: 10.1038/s41586-021-03403-8). This prior work should be acknowledged and discussed in the manuscript, particularly to contrast differences in experimental setup and to clarify how the current approach is uniquely suited to studying maternal responses to pup USVs.

2. The Results section still contains substantial portions of methodological description (lines 181-195, 199-205, 213-216). This text is either redundant with information already included in the Methods section or should be moved there.

3. The description of audio file processing provided in the response to reviewers should be incorporated into the Methods section to ensure reproducibility.

4. Information describing the "baseline" period used for fiber photometry recordings should be added to the manuscript.

5. The Wilcoxon signed-rank test is typically used when assumptions for parametric testing are not met. Please provide context in the manuscript explaining why this test was selected. In addition, the analysis should be described in the text rather than referring only to the figure, and it should be made explicit which data were analyzed using this approach (line 174).

Minor comments

1. Abstract, line 9: Please be more specific about the results (e.g., "during the pup contact phase of pup retrieval"). In addition, please consider including other key findings (e.g., lack of association with the number of pup calls).

2. Lines 56-57: Please provide a citation to support this statement.

3.Line 95: Please clarify what is meant by "natural" mating and what alternative conditions are being contrasted.

4. Lines 105-106: Please provide context for why the AAV was injected only into the left auditory cortex.

5. Lines 128-129: Please explain in the text which "acoustic features" and which aspects of "behavioral context" were analyzed, and how they were used. Because this is a methods-focused paper, sufficient detail should be provided for replication.

6. Line 134: Please describe the "synchronized camera system" in the Methods section.

7. Line 137: Prescreening dams may introduce bias in the animals selected for testing. This limitation should be acknowledged in the Discussion.

8. Lines 148-149: The number of trials per animal per day is still not reported and should be explicitly stated.

9. Line 184: Please remove the claim of "days-long monitoring," as such recordings were not performed in this study. The volume of neural, auditory, and video data generated by multi-day recordings is non-trivial and would require data storage and management infrastructure not described here. Longer recording durations could instead be mentioned as a potential future application in the Discussion, with appropriate citations.

10. Lines 242, 245, 251, and 252: Please report supporting statistics in the text.

11. Lines 249-251: Please provide additional information on the frequency of "other" maternal behaviors (e.g., total duration, number of bouts per session, and mean bout duration).

12. Grooming, crouching, and nest-building behaviors may not be fully expressed during a maternal retrieval task compared to conditions where the entire litter is present in the home cage. For example, tactile input from pups facilitates crouching behavior. Please discuss this limitation when interpreting results related to these "other" maternal behaviors.

13. Line 258: Please clarify whether recordings were performed under visible light, infrared illumination, or both. If only one condition was used, please describe the system. Alternative lighting conditions could be mentioned as a potential use case.

14. Line 310: Please replace "device" with "system."
