# Supplementary figures and images for: Simultaneous Recordings of Neuronal Activities, Pup Calls, and Maternal Behaviors in Lactating Mice
Source: eNeuro. 2026 Jul 28;13(7):ENEURO.0040-25.2026. doi: 10.1523/ENEURO.0040-25.2026 (PMC13431271; doi:10.1523/ENEURO.0040-25.2026)

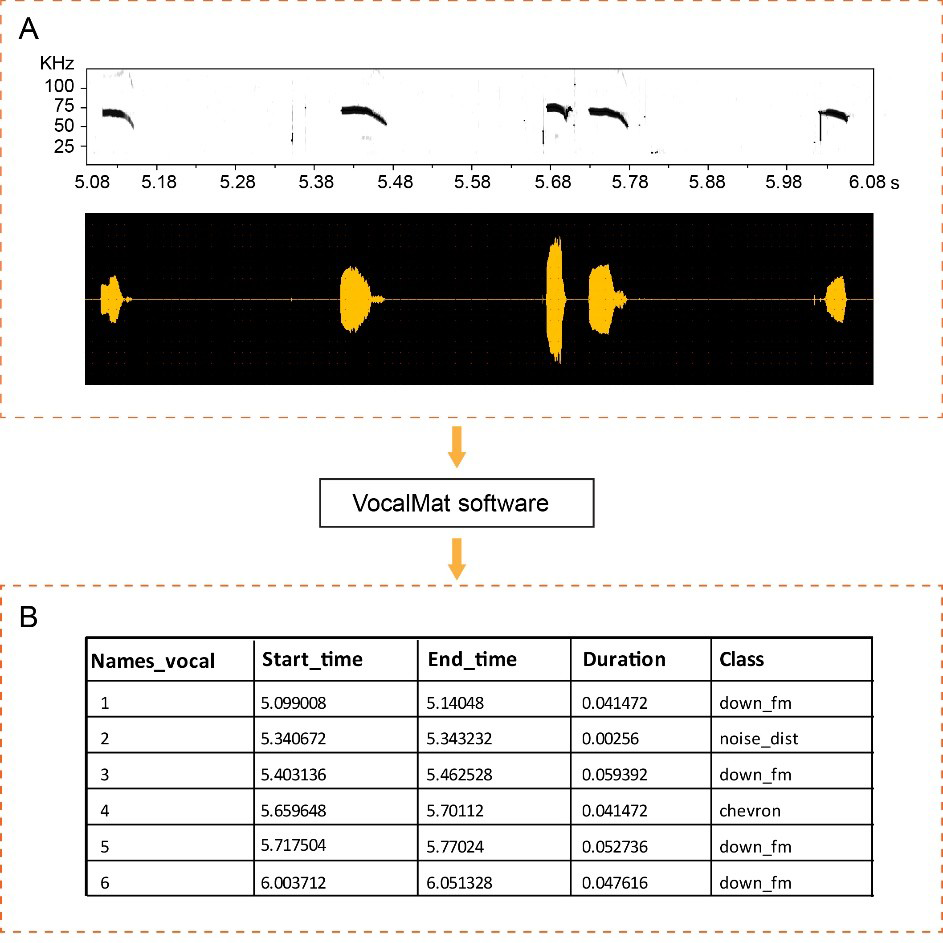

Supplement: Figure 1-1 — Schematic of pup calls analysis. Raw data (A) of pup calls obtained from Avisoft and the corresponding cleaned, classified output (B) generated by VocalMat. Figure Contributions: Yueling Zang, Jiechang Tang made the experimental device. Download Figure 1-1, TIF file. [file eneuro-13-ENEURO.0040-25.2026-s001.tif]

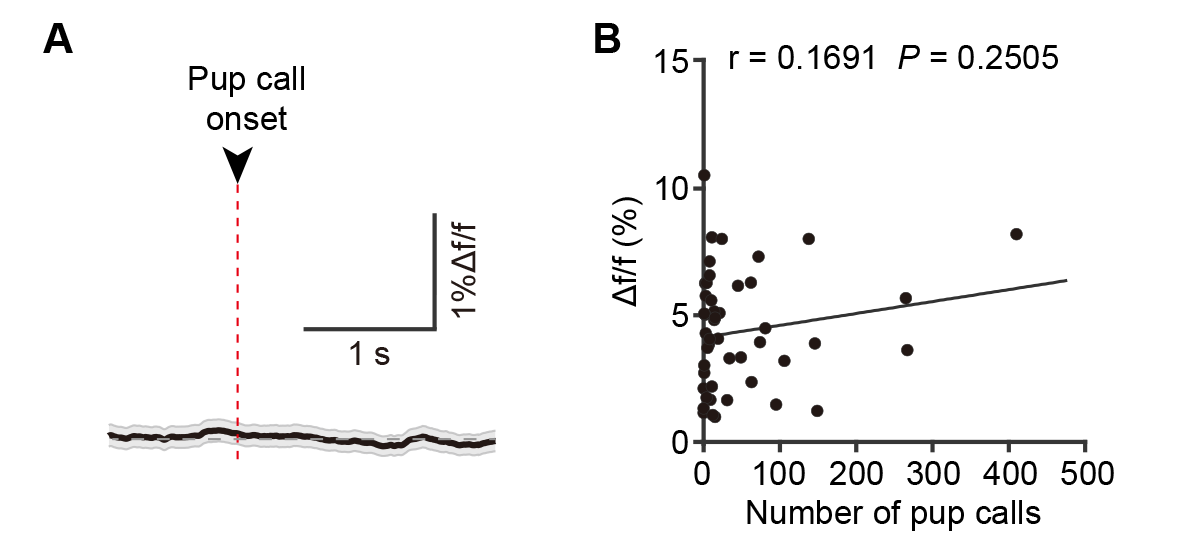

Supplement: Figure 4-1 — No significant AuCx responses to pup calls. A, Plot of Ca2+ transients aligned to the pup call onset (red dashed lines) of AuCx cells (n = 49 trials, N = 10 mice). B, No significant correlation between AuCx activation and the number of pup calls during pup contact (n = 48 trials, N = 10 mice). Figure Contributions: Huanhuan Wang, Yueling Zang performed the experiments; Jiechang Tang, Xia Wang, and Shanshan Liang analyzed the data. Download Figure 4-1, TIF file. [file eneuro-13-ENEURO.0040-25.2026-s002.tif]
